# Metabarcoding Reveals Diversity of Potentially Toxic Algae in Papeete Port (Tahiti)

**DOI:** 10.3390/toxins17080424

**Published:** 2025-08-20

**Authors:** Sara Fernandez, Lucie Cartairade, Eva Garcia-Vazquez, Serge Planes

**Affiliations:** 1 Department of Functional Biology, Faculty of Medicine, University of Oviedo, C/Julian Claveria s/n, 33006 Oviedo, Spain; fernandezfsara@uniovi.es; 2 USR3278 CRIOBE EPHE-CNRS-UPVD, 66860 Perpignan, France; lcartair@genoscope.cns.fr (L.C.); planes@univ-perp.fr (S.P.)

**Keywords:** harmful algae, health risks, seafood resource risks, metabarcoding, French Polynesia

## Abstract

Harmful algae are transported in various compartments of maritime vessels, making ports with heavy maritime traffic potential hotspots for their introduction and spread. In this study, we investigate the port of Papeete (Tahiti, French Polynesia), a key hub for numerous South Pacific shipping routes. Using metabarcoding on DNA extracted from water samples (environmental DNA, eDNA) we identified 21 species of harmful algae comprising to Bacillariophyceae (4), Dinophyceae (14), and Haptophyta (3 species). Three of those species are directly associated with fish mortality events without recognized toxigenic capacity. The remaining harmful algae species are known to produce a wide range of toxins, like the ciguatoxin produced by endemic *Gambierdiscus* sp., domoic acid, haemolysins, yessotoxins, and others. Health risks such as ciguatera and paralytic shellfish poisoning were identified. An increase in *Gambierdiscus* frequency in Papeete port waters was parallel to an increase in ciguatera fish poisoning events in Tahiti, which suggests the value of eDNA analysis for early warning of harmful algae presence. Management measures, including banning fishing near the ports, could prevent public health risks associated with harmful algae blooms.

## 1. Introduction

Harmful algae are toxin-producing algae capable of excessive proliferation in aquatic environments, a phenomenon known as harmful algae blooms (HABs, thereafter). Inadvertent consumption of these algae, typically through contaminated seafood, can cause severe health effects, and may even be lethal in some cases, depending on the type and amount of toxin ingested [1]. Given the increasing global occurrence of HABs, their detection and prediction are especially important. Therefore, monitoring harmful algae has been enhanced in recent years [2].

In the central and south Pacific Ocean, most reported HAB events are linked to seafood toxins, principally due to ciguatera [2]. Ciguatera fish poisoning (CFP) is caused by the dinoflagellate species of the genus *Gambierdiscus* and has been documented in Hawaii and other Pacific islands, with incidence rates reaching up to 100,000 cases annually [3]. In French Polynesia, consumption of seafood contaminated with ciguatoxins represents a major public health issue [4]. CFP incidence rates are dangerously high in some archipelagos [5], for example, on Rapa Island in the Australes [6], with a hotspot of *Gambierdiscus* species reported in Tahiti [5,7].

Harmful algae can be transported in ballast water [8], in bilge water [9], or even via biofouling on ship hulls [10], making ports potential entry points and reservoirs that should be surveyed as possible sources of these organisms. Conventional harmful algae surveys are based on collecting water samples and scanning them for microalgae using a light microscope; in addition, or as an alternative, molecular tools are also employed, providing a high sample throughput rate and sensitivity [11]. A method of harmful algae detection that is increasingly employed involves metabarcoding on environmental DNA (eDNA), that is, DNA extracted directly from water or sediment filter samples. Metabarcoding consists of amplifying from that DNA a sequence that serves to identify species, i.e., DNA barcode, followed by massive sequencing (Next Generation Sequencing, NGS) and bioinformatic analysis to compare sequences against reference databases for taxonomic identification [12,13]. Metabarcoding of water and sediment samples has been used to detect harmful algae in multiple studies [8,14].

**Figure 1:**
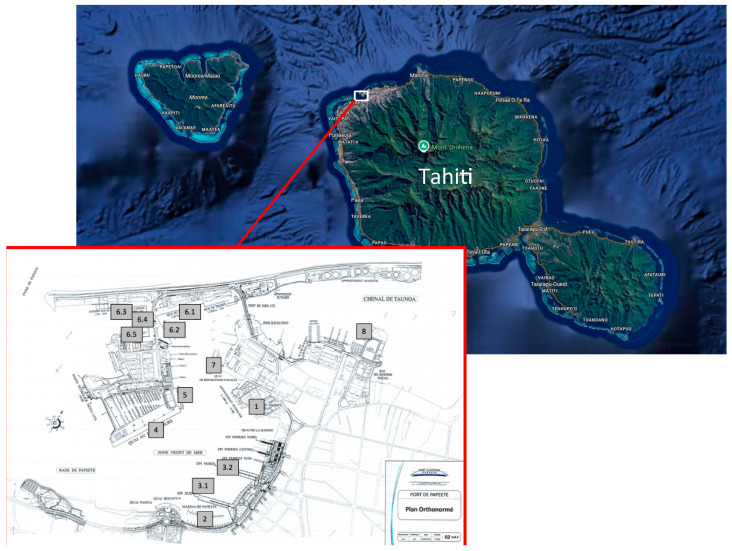
Map of Tahiti Island showing Papeete port. Modified from Google (n.d.). Retrieved on 28 June 2025. The location of the sampling points is shown in the map of the port (shown above on the left). The numbers (1, 2, 3.1, 3.2, 4, 5, 6.1 to 6.5, 7, 8) are the codes of the sampling points.

In this study, we applied eDNA metabarcoding on water samples from Papeete port (Tahiti). Papeete, the capital of French Polynesia, hosts the major port of the whole region (−17.5333°/−149.5666° coordinates; Figure 1) with facilities for general, bulk, and oil cargo, handling approximately 1,900,000 t of international cargo and 2,940,000 passengers annually [15]. Given this high volume of maritime traffic, we expected to find a relatively high number of toxic algae, particularly *Gambierdiscus* species. The objectives of this study were therefore (1) to inventory algae classified as harmful and (2) to assess the potential health risks of these species based on the toxins they are known to produce. To examine the relationship between the prevalence of harmful algae detected from metabarcoding and the reported toxicity outbreaks reported in the region, we compared the results obtained in 2018 [16] and 2023 (present study) for *Gambierdiscus* and CFP.

## 2. Results

The raw metabarcoding data (high-throughput sequencing, HTS) are available in NCBI database with the Bioproject number PRJNA1282046. A total of 1,738,896, 2,464,840, and 3,664,168 quality reads of the 18S rRNA, RBCL, and COI genes, respectively, were obtained from water samples taken from 13 sampling locations evenly distributed across Papeete port to have a good coverage of its area (Supplementary Table S1, where the letter S in the sample codes refers to samples taken from the surface and the letter F to deeper samples). Shallow (surface) samples provided 48.8%, 50.4%, and 51.6% of the quality sequences, respectively, for 18S rRNA, RBCL, and COI genes. Of the total quality reads, 1,501,192 were taxonomically assigned to 191 phytoplankton species (Supplementary Table S2), while 3697 (0.25%) corresponding to 65 phytoplankton taxa could not be assigned down to a species level. The taxonomic composition (Table 1) shows a higher proportion of diatoms (Bacillariophyta), followed by dinoflagellates (Myzozoa), both in terms of the number of species and the quality sequences assigned to them.

In total, 21 species known to be associated with HABs [16] were found, with the majority being dinoflagellates (14 species), followed by diatoms (4 species), and 3 Haptophyta species (Table 2). The proportion of species associated with HABs over the total number of phytoplankton species ranged between 0.147 and 0.207 across the sampling sites. The sites were homogeneous for this distribution of potentially harmful algae (contingency chi-square χ^2^ = 1.119, 12 degrees of freedom, *p* = 0.99).

Many species were detected across all the sampling locations, whereas *Amphidinium carterae* and *Gambierdiscus carpenteri* were less frequent, each found only from at only a single location. Three species (*Margalefidinium fulvescens*, *Margalefidinium polykrikoides*, and *Tripos fusus*) are known to be associated with fish mortality events but not with specific toxicogenic activity [17,18,19]. The remaining species are well-documented toxin producers [17]. Those of the genus *Pseudo-nitzschia* produce domoic acid; *Akashiwo sanguinea*, *Amphidinium carterae*, and *Pheaocystis globosa*, hemolytic toxins; *Alexandrium affine*, paralytic shellfish poisoning toxins; *Amphidoma* and *Azadinium* species, azaspiracids; *Coolia canariensis* and *Protoceratium reticulatum*, yessotoxins; *Gambierdiscus* species, maitotoxins and ciguatoxins (CFP toxins); *Ostreopsis ovata*, ovatoxins; *Chrysochromulina leadbeateri*, sterolysins; and *Prymnesium polylepis*, prymnesins (Table 2).

**Figure 2:**
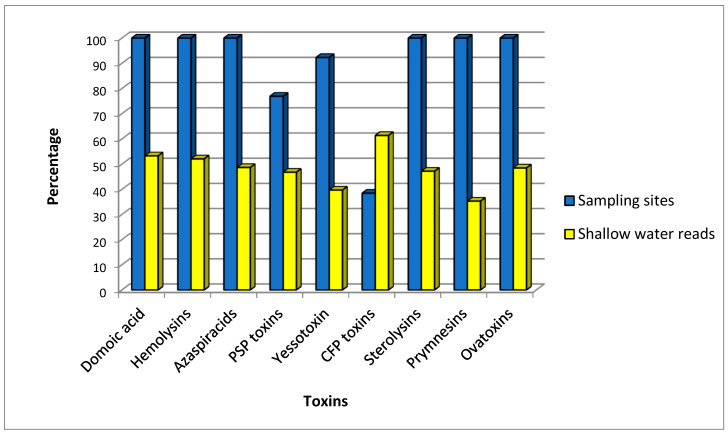
Frequency and depth of toxin producer species in Papeete port, measured, respectively, from the percent of sampling locations with the presence of species producing a toxin, and the proportion of reads found from surface water samples. PSP, paralytic shellfish poisoning; CP, ciguatera poisoning.

Based on the predominant toxin produced by each species and the frequency of the species in the port, our results indicate that producers of the majority of toxins were potentially present throughout the entire port area, with 53.2% of reads from shallow water and the remainder from deep water samples, showing a fairly balanced distribution across the water column (Figure 2). However, it was not equal for all the toxin producers. Notable exceptions included CFP toxins producers, only found in five sampling locations and mainly in shallow-water samples (61.4% of the reads); PSP toxins producers, present in 10 locations (46.8% reads from shallow samples); and yessotoxin and prymnesin producers, found at most or all sampling locations in deeper waters (39.7% and 35.3% reads, respectively, from shallow samples).

The threats of the harmful algae found from Papeete port in Tahiti are summarized in Table 3, based on the effects of the toxins they produce [20,21,22,23,24,25,26,27,28,29,30,31,32,33], the species affected, and known carriers of toxin producers. The effects range from neurotoxicity and inflammation to increased mortality, affecting from shellfish, fish resources, and humans. Carriers of toxin-producing algae are primarily shellfish and fish, but also crustaceans (Table 3).

**Figure 3:**
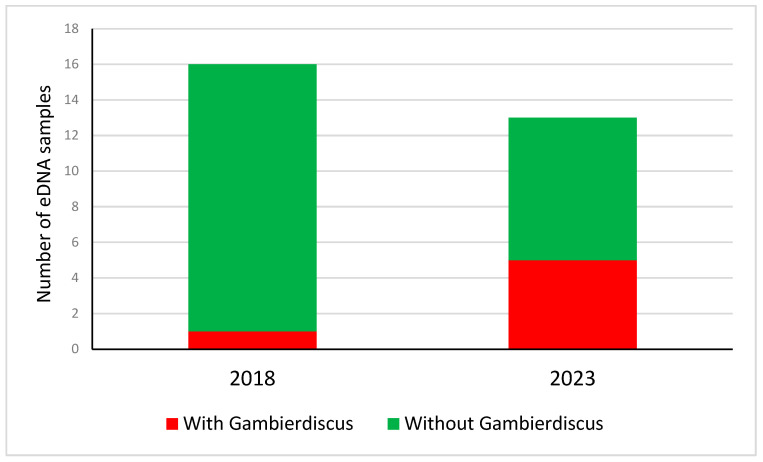
Number of eDNA samples from Papeete port with or without detected *Gambierdiscus* eDNA in 2018 [16] and 2023 (this study).

Several harmful algae found in this study had been found from metabarcoding in Papeete port in 2018 [16]: *Coolia canariensis*, *Protoceratium reticulatum*, *Margalefidinium polykrikoides*, and *Gambierdiscus*. Five out of the 13 Papeete port samples contained *Gambierdiscus* eDNA in our study in 2023 (Figure 3). In contrast, only one of the 16 samples analyzed from Papeete port in 2018 contained eDNA of *Gambierdiscus* [16]. In the two studies, *Gambierdiscus* eDNA was detected with the same 18S rDNA marker, thus a bias due to the marker is unlikely. The risk difference between 2018 and 2023 was 0.406 (95% confidence 0.129–0.842), being statistically significant (z = 0.13 with *p* = 0.033), suggesting an increase in the prevalence of species causative of CFP in 2023.

**Figure 4:**
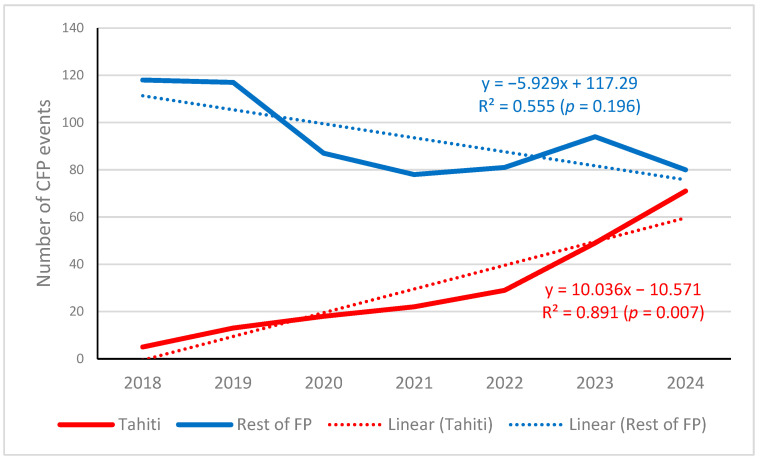
Number of ciguatera fish poisoning (CFP) events in Tahiti Island and in the rest of French Polynesia (FP) islands from 1 January 2018 to 31 December 2024. Graph generated by the authors using the data downloaded from the HAEDAT database for French Polynesia in the indicated period, filtering by Tahiti reports (red line) or without Tahiti (blue line).

In French Polynesia, the official data of harmful algae events are recorded from the Ciguatera Fish Poisoning Monitoring Program. In the period 2018–2024, a total of 655 events were recorded in all the islands of the whole region, of which 278 occurred in Tahiti Island. The evolution of the events along the studied period is presented in Figure 4. The difference between Tahiti and the rest of islands (excluding Tahiti) in the distribution of CFP events along the seven years considered was highly significant (χ^2^ = 20.17, 6 d.f., *p* = 0.003). It can be observed that, unlike in the rest of islands in the region, in Tahiti, the events increased noticeably in 2023 and even more in 2024 (5 events in 2018, followed by 13 in 2019 versus 49 events in 2023, followed by 71 in 2024). This coincides with the respective prevalence of *Gambierdiscus* species in Papeete port in 2018 and 2023 (see Figure 3).

## 3. Discussion

The detection of 21 potential HAB producers within Papeete port supports the hypothesis that ports act as hubs of harmful microalgae that are transported in different boat compartments [8,9,10]. Several species that pose serious toxicity risks for human health were present at all sampling sites, like the domoic acid producers *Pseudo-nitzschia cuspidata* and *P. galaxiae* [22], the azaspiracids producers *Azadinium* and *Amphidoma* [20], or *Ostreopsis ovata*, which produces ovatoxins [25]. Other locations outside the port should be sampled to confirm this hypothesis.

Two *Gambierdiscus* species associated with CFP were identified at five locations. This relatively low diversity could be unexpected given that Tahiti islands are considered hotspots of *Gambierdiscus* with seven known species of this genus [5], but these two species were the only detected in Papeete port in previous studies using a similar metabarcoding methodology [34], thus they would be stable in the port. The metabarcoding methodologies employed in previous studies on Papeete port [16,34] are not entirely comparable with the present study, principally due to different water volumes oscillating between 3 L [16] and 700 L [34] per replicate. Nevertheless, finding the same two species (28.6% of the *Gambierdiscus* described in all the island) in a single port using different water volumes in different years is already noticeable and points at the reproducibility of the results over time. Although the two species found in this study, *G. carpenteri* and *G. pacificus*, were not highly toxic in previous Tahitian reports [5], the risk of CFP remains if fish around the port are consumed.

The coincidence between the prevalence of *Gambierdiscus* in Papeete port measured from eDNA and the number of CFP events in Tahiti in 2018 and 2023, although cannot be taken as a direct cause-effect in absence of toxin studies, could be an indirect indicator of the validity of the eDNA as an early alert system of harmful algae presence, supporting other studies [8,14]. These results suggest an increase in CFP agents in Papeete port, perhaps due to the introduction of new species and strains of harmful algae via maritime traffic over the years, something that should be studied more carefully including toxin analysis in the *Gambierdiscus* found in the port for comparison with toxins found in CFP outbreaks in Tahiti.

With respect to the potential threats of the harmful algae found in Papeete port in Tahiti (Table 3), their potential toxins indicate threats to both human health and seafood resources [20,21,22,23,24,25,26,27,28,29,30,31,32]. Azaspiracids, produced by *Azadinium* and other dinoflagellates [20], are known to cause azaspiracid poisoning (AZA), which manifests as nausea, vomiting, diarrhea, and other gastrointestinal symptoms. CFP toxins (ciguatoxin, maitotoxin) cause a range of symptoms that include neurological, gastrointestinal, and cardiovascular problems in humans, and reduced recruitment in fish [21]. The domoic acid is a neurotoxin that affects humans and other vertebrates like seabirds and marine mammals, causing amnesic shellfish poisoning characterized by short-term memory loss and brain damage [22]. Hemolysins reduce survival of affected fish [23] and shellfish resources, including pearl oysters [24]. Ovatoxins [25] present in marine aerosols produce inflammatory response by inhalation, causing diverse symptoms like rhinorrhea, eye and nose irritation, and general malaise in humans. Prymnesins are toxic metabolites that deter grazing in herbivore fish and have cytotoxic effects in fish and shellfish, reducing their survival; they also harm bivalve predators like seabirds [26,27]. Paralytic shellfish poisoning (PSP) in humans is caused by neurotoxins, principally saxitoxin, produced by *Alexandrium* species that are carried by shellfish but also by fish and crustaceans [28,29]. Sterolysins [30,31] produced by different microalgae cause recurrent fish kills. Finally, yessotoxins and their analogues are another type of toxins that causes mortality in different fish and shellfish species [32,33].

Some harmful algae that are ubiquitously detected in the port can cause mortality to fisheries resources, impacting both finfish and shellfish (Table 2). Those that reduce bivalve survival, like *Akashiwo sanguinea* [24], may be of special concern in French Polynesia for the risk that may pose to pearl oysters that are a key resource in the region [35]. Moreover, the five species detected in 2018 [16,34] and again in 2023 are probably established in Papeete port, as opposed to brief introductions. *Coolia canariensis*, *Protoceratium reticulatum*, *Margalefidinium polykrikoides*, and the two *Gambierdiscus* species commented on above would also be of special concern, because they could spread from the port that may act as a reservoir for them.

This study contributes to the growing body of work employing metabarcoding for harmful algae detection [8,14,36]. The method is reliable and efficient for the description of plankton communities when based on high-quality data [37], and using three metabarcodes, we are capturing the majority of phytoplankton diversity [38]. However, there is a limitation: with the metabarcodes here employed, we are identifying species but not the strains that produce toxins. The method could be improved, amplifying genes that code for the toxins, or that are able to recognize toxin-producing strains. This way the toxic load could be estimated in water samples. Another way of improving the accuracy of health danger evaluation in port ecosystems could be to combine metabarcoding surveys with direct measurements of toxins potentially produced by the species found from eDNA. More generally, routine checking of harmful algae in locally consumed seafood as well as in seafood exports would ensure safe and healthy consumption of French Polynesian fishing and aquaculture resources.

Future research directions should explore the diversity and the frequency of HAB producers in other maritime ports, and explore for methods to prevent the transport of microalgae by ships. Potential HAB mitigation methods include physical aeration to prevent blooms [39], clays to flocculate algal cells and their ichthyotoxins [40], or biological agents like bacteria and viruses to control the microalgae [41]. However, the methods developed so far remain partially efficient; more methods to inhibit the proliferation of HABs would be useful [42,43]. Promising methods based on cultivation of seaweeds in sites of interest that may reduce the lethal effects of some microalgae [44] could be explored. More importantly, methods to treat hulls and ballast water to avoid shipping transport of microalgae, like biocides, heat treatment, and others [45], should be further developed.

These findings strongly suggest that consuming fish and shellfish collected within or nearby the port may be a risk to human health. In French Polynesia, fishing is prohibited inside port areas, with angling being permitted only nearby [46]. In other regions illegal angling fishing within ports is reported as relatively frequent [47], it being not rare to see people angling from some piers within port areas, especially in small ports. Although this study focused on the presence of toxin-producer species rather than direct toxin measurements and recognizing that not all the strains are equally toxic [48], applying the precautionary principle, a ban of fishing nearby this and other ports, and a control of illegal angling within ports would be recommended.

Finally, here we have focused on port areas that are impacted by maritime transport. Harmful algae can also be naturally introduced in the South Pacific by oceanographic driving. An example is the expansion of *Pseudochattonella verruculosa* to the south due to changes in water column stratification associated with global climate change; its convergence with *A. catenella* caused a catastrophic HAB event in Chilean waters in 2016 [49]. It is difficult to estimate the relative weight of maritime transport and natural oceanographic shipping on the expansion of harmful algae, but from our results it seems that controlling port areas will help to control HABs. Regional early warning systems could combine molecular methods like metabarcoding, remote sensing, and oceanographic records.

## 4. Materials and Methods

### 4.1. Sampling and eDNA Extraction

Sampling was conducted between 22 May and 5 June of 2023. At each of 13 sampling sites within Papeete port, three replicates of 20 L water were collected from two depths (surface and 2 m above the substrate, ranging from 1 to 15 m depth) and using two filter pore sizes (1.2 and 0.2 µm) to capture as much diversity as possible. DNA was extracted from the filters using the Quick-DNA Fecal/Soil Miniprep Kit (Zymo Research, Irvine, CA, USA), with modifications optimized for marine samples. The protocol combined mechanical and chemical cell lysis followed by column-based purification and a final inhibitor removal step, ensuring DNA quality for downstream PCR amplification. The replicates were joined by sampling point and depth for obtaining a higher eDNA quantity.

DNA samples were purified using the Mag-Bind RXNPure Plus magnetic beads (Omega Biotek, Norcross, GA, USA), following the instructions provided by the manufacturer. Magnetic beads were added in 1.5 times the volume of the original DNA extract to retrieve the whole spectrum of DNA fragment sizes in the samples. The purified DNA was resuspended in 60 µL. We quantified the DNA concentration in each extract using the Qubit High Sensitivity dsDNA Assay (Thermo Fisher Scientific; BIOGEN Científica, Madrid, Spain).

### 4.2. DNA Metabarcoding Library Preparation and Sequencing

DNA metabarcoding library preparation and sequencing were performed by AllGenetics and Biology SL www.allgenetics.eu (accessed on 16 July 2025). DNA metabarcoding procedures followed Douard et al. [50], based on three libraries that are summarized next. The first one was constructed by amplifying a 365 bp fragment of the chloroplastic rcbL gene using a cocktail of primers [51]:

Forward

· Diat_rbcL_708F_1 (5′ AGGTGAAGTAAAAGGTTCWTACTTAAA 3′)

· Diat_rbcL_708F_2 (5′ AGGTGAAGTTAAAGGTTCWTAYTTAAA 3′)

· Diat_rbcL_708F_3 (5′ AGGTGAAACTAAAGGTTCWTACTTAAA 3′)

Reverse 

· R3_1 (5′ CCTTCTAATTTACCWACWACTG 3′)

· R3_2 (5′ CCTTCTAATTTACCWACAACAG 3′)

In the second library, a fragment of the mitochondrial COI gene of around 365 bp (including primer sequences) was amplified using the primers mICOIintF [52] as forward and jgHCO2198 [53] as reverse. In the third, the V4 domain of the nuclear 18S rRNA gene (460 bp) was amplified with the primers Uni18SF and Uni18SR [54].

All the primers had 5′ Illumina sequencing primer sequences attached. In the first amplification step, PCRs were carried out in triplicate in a final volume of 12.5 μL, containing 1.25 μL of template DNA, 0.5 μM of the primers, 3.13 μL of Supreme NZYTaq 2x Green Master Mix (NZYTech), and ultrapure water up to 12.5 μL. The reaction mixture was incubated with an initial denaturation step at 95 °C for 5 min, followed by 35 cycles of 95 °C for 30 s, annealing temperature for 45 s, 45 s, 72 °C for 45 s, and a final extension step at 72 °C for 7 min. Annealing temperatures were 47 °C, 51.4 °C, and 47.4 °C for the first, second, and third library, respectively. The products from the three PCR replicates were pooled together for the subsequent steps.

A second amplification step of 5 cycles and annealing at 60 °C was carried out, attaching oligonucleotide indices for multiplexing different libraries in the same sequencing pool [55]. Negative PCR controls without DNA were included in all the rounds. These blanks were also pooled together (BPCR) for library preparation.

The libraries were run on GreenSafe (NZYTech)-stained agarose gels (2%) to check the library size under UV light. Then, they were purified with Mag-Bind RXNPure Plus magnetic beads (Omega Bio-tek) and pooled in equimolar quantities using Qubit dsDNA HS Assay (Thermo Fisher Scientific) for quantification. A NovaSeq PE250 flow cell (Illumina) was employed for sequencing, aiming at two Gb of output for each marker.

### 4.3. Bioinformatics Analysis

Standard bioinformatics analysis was performed [50]. First, traces of adapter dimers were removed using Cutadapt v3.5 [56], and sequencing indices were trimmed after a demultiplexing step. FASTQ file quality was checked using FastQC [57]. The output was summarized using MultiQC [58].

Qiime2 2021.4 [59] was employed for further analyses. Primers were trimmed with the q2-cutadapt plugin [56]. DADA2 [60] was used for denoising via q2-dada2. To construct a local database, nucleotide sequences were downloaded from NCBI using the e-search queries “COI NOT environmental NOT unclassified sequences NOT bacteria”, “18S NOT uncultured NOT fungi NOT land plants NOT unclassified NOT unidentified NOT archaea NOT bacteria NOT viruses NOT genome”, and “rbcl NOT uncultured NOT algae NOT unclassified NOT genome” using Rescript within qiime2 [23].

The quality-filtered sequences were taxonomically assigned using the q2-feature-classifier [61] with an exigent assignation (97% as percent of identity and e-value below 10 × 10^−50^), for high confidence on the species status considering the metabarcodes employed.

An ASV (Amplicon Sequence Variant) table was constructed with the reads of each assigned species by sample, retaining those assigned at least to a genus level and eliminating DNA from humans and other terrestrial taxa. For downstream analysis, only those samples with >200 reads in the curated ASV table were considered. Taxonomic references and distribution of the species assigned from DNA were validated and confirmed using WoRMS [62] and the French National Inventory of Natural Heritage [63] databases.

### 4.4. Harmful Algae Events in French Polynesia

The harmful algae events recorded in French Polynesia for the period 2018–2024 were downloaded from the Harmful Algae Events Database HAEDAT [64]. The following search terms were employed: Country, French Polynesia; period years, 2018–2025; nature of the harmful event: any; organism affected: any; associated syndrome: any; species implicated in toxin transmission (transvector): any.

In the French Polynesian network for ciguatera surveillance, CFP cases are reported on a volunteer basis. The reporting forms [65] include the archipelago, island, and city/village as geographical information, but not the exact location within a city with coordinates. Thus, it is not possible to know if CFP events occurred within Papeete port.

### 4.5. Statistical Analysis

Statistics was carried out with the free PAST software v. 4.03 [66]. Distributions like the number of CFP events in the years considered in Tahiti and the rest of the French Polynesian islands were compared using contingency chi-square analysis. The prevalence of *Gambierdiscus* (detected from eDNA) in Papeete port was compared between 2018 and the current dataset based on frequency differences and *z* to estimate their significance. The standard *p* < 0.05 significance threshold was adopted.

## Figures and Tables

**Table 1:** Taxonomic summary of the phytoplankton found in this study from Papeete port using metabarcoding. Number of species and number of quality sequences per phylum are presented, combining the three markers.

Phylum	Species	Sequences
Bacillariophyta	115	881,323
Chlorophyta	5	18,852
Cryptophyta	3	133
Crysophyta	1	73
Haptophyta	4	1581
Heterokontophyta	1	3393
Myzozoa	52	125,937
Ochrophyta	10	69,793

**Table 2:** Total number of quality sequences (Reads) obtained from Papeete port for each potential toxin producer species [17]. The number of port sampling locations from which each species was found is indicated.

Class	Species	Main Toxins	Reads	Sampling Locations
Bacillariophyceae	*Pseudo-nitzschia caciantha*	Domoic acid	12	4
Bacillariophyceae	*Pseudo-nitzschia cuspidata*	Domoic acid	3395	13
Bacillariophyceae	*Pseudo-nitzschia delicatissima*	Domoic acid	62	12
Bacillariophyceae	*Pseudo-nitzschia galaxiae*	Domoic acid	694,147	13
Dinophyceae	*Akashiwo sanguinea*	Hemolysin-like	313	13
Dinophyceae	*Alexandrium affine*	PSP toxins	252	10
Dinophyceae	*Amphidinium carterae*	Hemolysins	13	1
Dinophyceae	*Amphidoma languida*	Azaspiracids	3056	13
Dinophyceae	*Azadinium poporum*	Azaspiracid	71	11
Dinophyceae	*Azadinium spinosum*	Azaspiracids	476	13
Dinophyceae	*Coolia canariensis*	Yessotoxin analogue	10	2
Dinophyceae	*Gambierdiscus carpenteri*	Maitotoxin	11	1
Dinophyceae	*Gambierdiscus pacificus*	Ciguatoxin- and MTX-like toxins	33	4
Dinophyceae	*Margalefidinium fulvescens*	Associated with fish kills without confirmed toxin production	130	12
Dinophyceae	*Margalefidinium polykrikoides*	Associated with fish kills without confirmed toxin production	2884	13
Dinophyceae	*Ostreopsis ovata*	Ovatoxins	683	13
Dinophyceae	*Protoceratium reticulatum*	Yessotoxin	48	11
Dinophyceae	*Tripos fusus*	Associated with fish kills, without confirmed toxin production	513	13
Haptophyta	*Chrysochromulina leadbeateri*	Sterolysin-like	254	13
Haptophyta	*Phaeocystis globosa*	Hemolysins	288	13
Haptophyta	*Prymnesium polylepis*	Prymnesins	119	13

**Table 3:** Summary of harmful effects of the toxins potentially produced by HABs found from Papeete port using metabarcoding. References are given for each toxin. AZP, azaspiracid poisoning; ASP, amnesic shellfish poisoning; PSP, paralytic shellfish poisoning; CP, ciguatera poisoning.

Toxin	Main Harmful Effects	Affected Species	Carriers of Producing Algae
Azaspiracids [20]	AZP	Humans	Shellfish, crabs
CFP toxins [21]	CFP, reduced recruitment	Humans, fish	Fish
Domoic acid [22]	ASP, neurotoxicity	Humans, birds, mammals	Shellfish, finfish
Hemolysins, hemolysin-like [23,24]	Increased mortality	Shellfish; finfish	Shellfish
Ovatoxins [25]	Inflammatory response	Humans	Direct contact
Prymnesins [26,27]	Increased mortality	Fish, shellfish, seabirds	Direct contact/shellfish
PSP toxins [28,29]	PSP	Humans	Shellfish, fish, crustaceans
Sterolysins, sterolysin-like [30,31]	Increased mortality	Fish	Direct contact
Yessotoxin and analogues [32,33]	Increased mortality	Fish, shellfish	Shellfish

## Data Availability

Data supporting reported results can be found in the NCBI SRA, with the bioproject number PRJNA1282046.

## Supplementary Materials

The following supporting information can be downloaded at: https://www.mdpi.com/article/10.3390/toxins17080424/s1. Table S1: DADA analysis of the Next Generation sequences obtained from Papeete port water samples; Table S2: List of phytoplankton species and the total number of sequences obtained from each sampling location within Papeete port.
