# Correction: Fernandez et al. Metabarcoding Reveals Diversity of Potentially Toxic Algae in Papeete Port (Tahiti). *Toxins* 2025, *17*, 424

**DOI:** 10.3390/toxins18010011

**Published:** 2025-12-24

**Authors:** Sara Fernandez, Lucie Cartairade, Eva Garcia-Vazquez, Serge Planes

**Affiliations:** 1 Department of Functional Biology, Faculty of Medicine, University of Oviedo, C/Julian Claveria s/n, 33006 Oviedo, Spain; fernandezfsara@uniovi.es; 2 USR3278 CRIOBE EPHE-CNRS-UPVD, 66860 Perpignan, France; lcartair@genoscope.cns.fr (L.C.); planes@univ-perp.fr (S.P.)

The authors realized there was an error in the original publication [1]. The preliminary version with cumulative (not annual) CFP events in Tahiti was used inadvertently. The corrected Figure 4 appears below.

A correction has been made to Section 2, Paragraph 7, accordingly:

“It can be observed that, unlike in the rest of islands in the region, in Tahiti, the events increased noticeably in 2023 and even more in 2024 (5 events in 2018, followed by 8 in 2019 versus 20 events in 2023, followed by 22 in 2024). This coincides with the respective prevalence of *Gambierdiscus* species in Papeete port in 2018 and 2023 (see Figure 3).”

The authors state that the scientific conclusions are unaffected. This correction was approved by the Academic Editor. The original publication has also been updated.

## Figures and Tables

**Figure 4:**
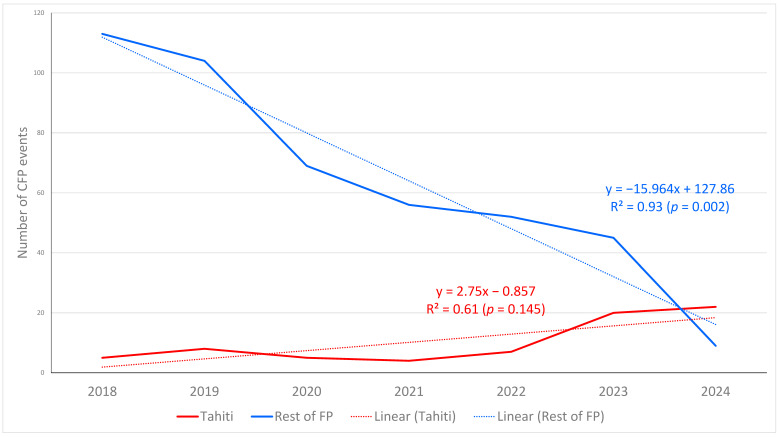
Number of ciguatera fish poisoning (CFP) events in Tahiti Island and in the rest of French Polynesia (FP) islands from 1 January 2018 to 31 December 2024. Graph generated by the authors using the data downloaded from the HAEDAT database for French Polynesia in the indicated period, filtering by Tahiti reports (red line) or without Tahiti (blue line).
